# Antagonistic roles of Nibbler and Hen1 in modulating piRNA 3′ ends in *Drosophila*

**DOI:** 10.1242/dev.128116

**Published:** 2016-02-01

**Authors:** Hui Wang, Zaijun Ma, Kongyan Niu, Yi Xiao, Xiaofen Wu, Chenyu Pan, Yun Zhao, Kai Wang, Yaoyang Zhang, Nan Liu

**Affiliations:** 1 Interdisciplinary Research Center on Biology and Chemistry, Shanghai Institute of Organic Chemistry, Chinese Academy of Sciences, Shanghai 201203, China; 2 State Key Laboratory of Cell Biology, Institute of Biochemistry and Cell Biology, Shanghai Institutes for Biological Sciences, Chinese Academy of Sciences, Shanghai 200031, China; 3 Zilkha Neurogenetic Institute, University of South California, Los Angeles, CA 90033, USA; 4 Collaborative Innovation Center of Chemistry for Life Sciences, Shanghai Institute of Organic Chemistry, Chinese Academy of Sciences, Shanghai 200032, China

**Keywords:** *Nbr*, *Hen1*, piRNA, 3′ terminal trimming, 2′-*O*-methylation, Transposon, Small RNA sequencing, *Drosophila*

## Abstract

In eukaryotes, aberrant expression of transposable elements (TEs) is detrimental to the host genome. Piwi-interacting RNAs (piRNAs) of ∼23 to 30 nucleotides bound to PIWI clade Argonaute proteins silence transposons in a manner that is strictly dependent on their sequence complementarity. Hence, a key goal in understanding piRNA pathways is to determine mechanisms that modulate piRNA sequences. Here, we identify a protein-protein interaction between the 3′-to-5′ exoribonuclease Nibbler (Nbr) and Piwi that links Nbr activity with piRNA pathways. We show that there is a delicate balance in the interplay between Nbr and Hen1, a methyltransferase involved in 2′-*O*-methylation at the 3′ terminal nucleotides of piRNAs, thus connecting two genes with opposing activities in the biogenesis of piRNA 3′ ends. With age, piRNAs become shorter and fewer in number, which is coupled with the derepression of select TEs. We demonstrate that activities of *Nbr* and *Hen1* inherently contribute to TE silencing and age-dependent profiles of piRNAs. We propose that antagonistic roles of *Nbr* and *Hen1* define a mechanism to modulate piRNA 3′ ends.

## Abstract (precis)

**Summary:** Antagonism between Nbr and Hen1 represents a novel mechanism for the modulation of piRNA sequences, revealing new players involved in the silencing of transposable elements.

## INTRODUCTION

Transposable elements (TEs) are abundant in eukaryotes and their aberrant expression and transposition can have deleterious effects on the host genome (Levin and Moran, 2011). Piwi-interacting RNAs (piRNAs), which comprise the largest class of small non-coding RNAs in gonadal cells, are involved in silencing the expression of TEs, safeguarding genome stability (Malone and Hannon, 2009; Siomi et al., 2011). Given their central importance, the biogenesis of piRNAs and modulation of piRNA pathways are areas of broad interest.

Compared with other endogenous small non-coding RNAs, such as small interfering RNAs (siRNAs, ∼21 nt) (Elbashir et al., 2001) and microRNAs (miRNAs, ∼22 nt) (Tomari et al., 2007), piRNAs are specifically germline enriched and show a much broader size range of ∼23 to 30 nt, suggesting a unique biogenesis mechanism (Aravin et al., 2006). In *Drosophila*, according to the mechanistic cascades by which they are produced, piRNAs can be divided into three types: primary, secondary and tertiary (Siomi and Siomi, 2015). Primary piRNAs are produced from long precursory transcripts derived from one of 142 such piRNA clusters – discrete genomic loci comprising complex structures of transposon remnants (Brennecke et al., 2007). Zucchini (Zuc), an endonuclease, mediates cleavage to produce piRNAs that tend to begin with 5′ uridine (1U) (Brennecke et al., 2007; Ipsaro et al., 2012; Nishimasu et al., 2012). Primary piRNAs are bound to Piwi or Aubergine (Aub). Aub-bound primary piRNAs cut active TE mRNAs to yield secondary piRNAs bound to Ago3, which in turn cleave original precursory piRNAs to generate new Aub-bound piRNAs, thus forming the so called ping-pong cycles that amplify piRNAs (Brennecke et al., 2007; Gunawardane et al., 2007; Li et al., 2009). Tertiary piRNAs are derived from a Zuc-dependent phased cleavage along precursor piRNAs following the initiation sites where Ago3-bound secondary piRNAs trigger the cut. Tertiary piRNAs are bound to Piwi (Han et al., 2015a; Mohn et al., 2015). Collectively, these mechanisms of biogenesis are mutually dependent and allow the production of extraordinarily abundant and diverse piRNA sequences that are ready to destroy transposon mRNAs when aberrantly expressed.

Despite recent progress, piRNA pathways are only beginning to be revealed. Previous reports have addressed mechanistic frameworks by which piRNAs can be made, but genes that modulate piRNA sequences are poorly understood. Intriguingly, most individual piRNAs are of low abundance, but one prominent testicular piRNA, AT-chX-1, has multiple isoforms with defined sequences (Nishida et al., 2007). Deep sequence analysis of piRNAs from testis reveals that AT-chX-1 all begin with 1U, followed by heterogeneous 3′ ends, descending by single nucleotides. Based on established models, Zuc cleavage is tentatively involved to liberate the 5′ end of AT-chX-1, given the 1U bias, but factors dictating the modulation of 3′ ends have not been well specified. This raises the possibility that a putative exoribonuclease might be involved to trim piRNA from the 3′ end. Using silkworm and *in vitro* biochemistry, a study predicted an active processing that determined the 3′ end formation of piRNAs (Kawaoka et al., 2011). Similarly, a recent report proposed roles of Nbr, an established 3′-to-5′ exoribonuclease previously described in *Drosophila* miRNA pathways, in piRNA biogenesis (Feltzin et al., 2015). The extent to which Nbr trims piRNA populations, including AT-chX-1, and whether this modulation is functionally relevant in the repression of TEs, remain uncharacterized.

A common feature of piRNA 3′ ends is 2′-*O*-methylation, a chemical modification catalyzed by Hen1, a methyl transferase (Horwich et al., 2007; Saito et al., 2007). Studies in *Arabidopsis* have implicated a protective effect of 2′-*O*-methylation (Li et al., 2005; Yu et al., 2005). Since virtually all fly piRNAs are 2′-*O*-methylated, if that effect holds true in *Drosophila* then such a protective mechanism should have a much broader impact on piRNAs than previously estimated. However, it remains to be determined how *Hen1* impacts fly piRNAs at the genome level. In animals, the progression of normal aging is coupled with a functional deterioration in multiple systems, including fertility (Lopez-Otin et al., 2013). Provocatively, aberrant induction of TEs has been noted in aging brains, indicating a late-onset decline in the control of TEs (De Cecco et al., 2013). But whether and how piRNA pathways are modulated with age are poorly studied. Here, we interrogate *in vivo* functions of *Nbr* and *Hen1*, and dissect antagonistic roles between these two genes that profoundly impact piRNA 3′ ends in *Drosophila*. We further extend our findings into the chronic modulation of piRNA pathways that relates to an age-dependent activity of *Nbr*.

## RESULTS

### Endogenous Nbr is ovary enriched and interacts with Piwi

**Fig. 1.**
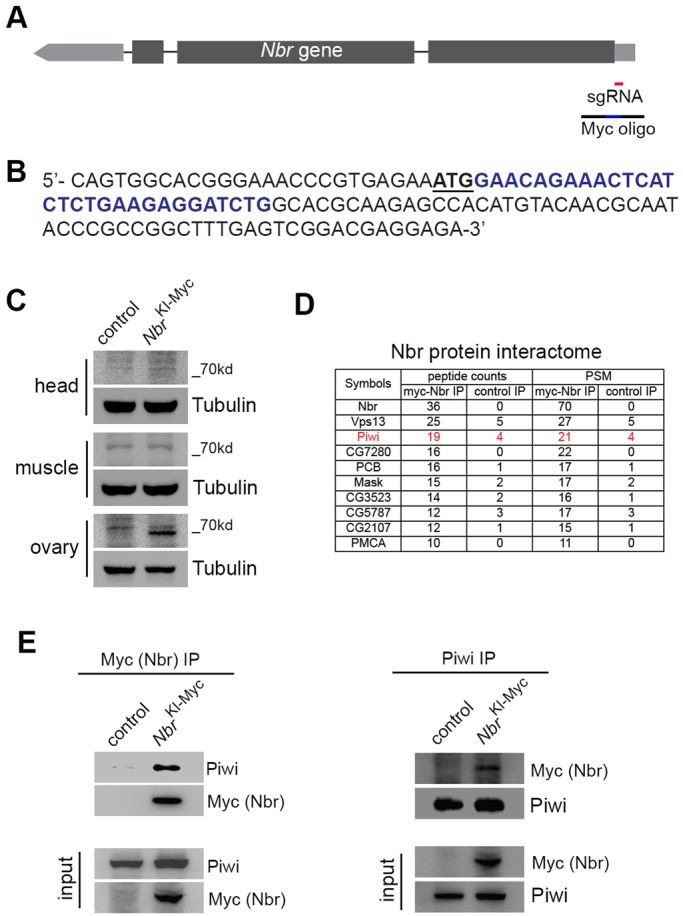
**Endogenous Nbr is ovary enriched and interacts with Piwi.** (A) The *Drosophila* *Nbr* locus and guiding RNAs used to make *Nbr*^KI-Myc^. To produce an *Nbr* Myc tag knock-in allele using the CRISPR/Cas9 method, sgRNA (red) was co-injected with a DNA oligonucleotide (black line, interrupted with blue representing Myc coding sequence). (B) In *Nbr*^KI-Myc^, the *Nbr* gene contains sequence (blue) encoding the Myc tag inserted after the *Nbr* ATG start codon (underlined). Resulting *Nbr*^KI-Myc^ flies were backcrossed to the control homogeneous background for five generations to ensure background clearance. (C) Nbr is ovary enriched. In adult flies, a protein signal corresponding to Nbr protein on the western blot could be clearly seen for the ovary, but not head or muscle. Genotypes: control (5905) and *Nbr*^KI-Myc/KI-Myc^ (*Nbr*^KI-Myc^). (D) Endogenous protein interactome determines that Nbr interacts with Piwi. Using endogenous Nbr as bait for immunoprecipitation and mass spectroscopy analysis, Piwi (red) was captured among the top-ranked interacting proteins. Ranking was based on peptide counts. Highly stringent criteria were applied, including peptide counts greater than 10 and PSM fold change greater than 5. Proteins were from ovaries. Genotypes as in C. (E) Co-IP experiment confirming that Piwi interacts with Nbr. Proteins were from ovaries. Genotypes as in C.

To study the *in vivo* function of *Nbr*, we characterized the Nbr interactome based on its endogenous protein-protein interaction. We utilized the CRISPR/Cas9 method in *Drosophila* (Ren et al., 2013; Yu et al., 2013). We designed a single-stranded oligodeoxynucleotide repair template that includes a 30 nt Myc tag sequence flanked by 146 nt homology arms corresponding to the *Nbr* genomic sequence (Fig. 1A). Resulting flies expressed a Myc tag fused in frame within the N′ terminus of the Nbr protein (Fig. 1B), hereafter termed *Nbr*^KI-Myc^. *Nbr*^KI-Myc^ flies showed a normal miR-34 trimming pattern, suggesting that the addition of a Myc tag has no effect on native protein function (data not shown). Western blot showed that Nbr protein is relatively highly expressed in ovaries compared with other adult tissues (Fig. 1C).

To study the Nbr interactome, we dissected ovary tissues and performed Nbr protein immunoprecipitation using a Myc antibody pull-down followed by mass spectrometry. To ensure stringency and quantification, we ranked the result list with more than ten peptide hits, and narrowed it down to those with peptide-spectrum match (PSM) counts greater than 5-fold enrichment. Strikingly, this approach found Piwi among the top hits (Fig. 1D). To confirm this finding, we generated a polyclonal antibody for the *Drosophila* Piwi protein. We first tested the specificity of this new antibody using *piwi* mutant flies that we made via the CRISPR/Cas9 method, hereby named *piwi*^cas9^ (Fig. S1A-C). Then, using this specific Piwi antibody and endogenous protein co-immunoprecipitation assay (co-IP), we detected Piwi when pulling down Myc-Nbr; reciprocally, we found Nbr in the Piwi pull-down (Fig. 1E). Together, these assays established endogenous protein-protein interaction. We next used *Drosophila* Schneider 2 cells and co-IP to determine if Nbr can interact with other Piwi family proteins. Our data showed that Nbr appears to strongly interact with Piwi, but not Aub or Ago3 (Fig. S1D). Notably, it has been known that Piwi is a hallmark protein involved in virtually all major steps of piRNA pathways. Thus, association with Piwi might link Nbr function with piRNAs.

### Nbr trims piRNAs from both germline and somatic tissues

**Fig. 2.**
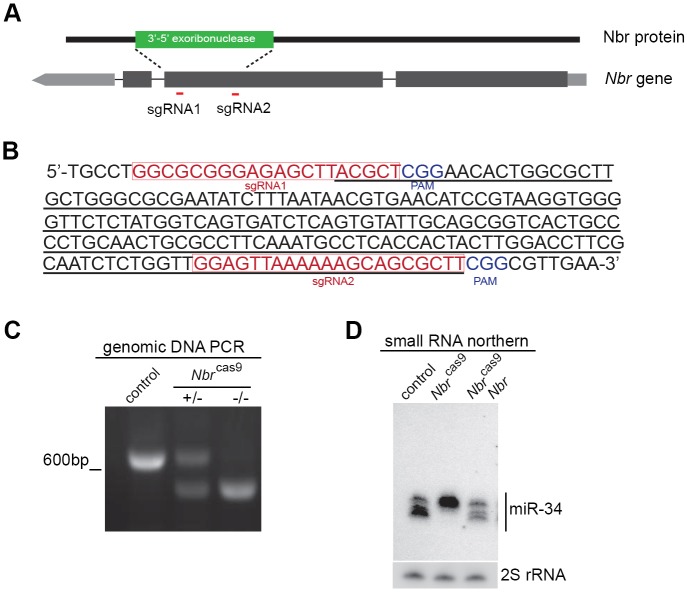
**Generation of a new *Nbr* loss-of-function allele.** (A) The *Nbr* locus and guiding RNAs used to make *Nbr*^cas9^. To produce an *Nbr* loss-of-function allele using the CRISPR/Cas9 method, sgRNA1 and sgRNA2 (red lines) were used to induce site-specific deletion of the 3′-to-5′ exoribonuclease domain of Nbr. (B) Part of the *Nbr* genomic sequence illustrating features of the CRISPR/Cas9-mediated *Nbr*^cas9^ loss-of-function allele. sgRNAs and protospacer adjacent motifs (PAMs) are highlighted. Nucleotides deleted are underlined. Resulting *Nbr* mutant flies were backcrossed to the control homogeneous background for five generations to ensure background clearance. (C) PCR analysis confirms that *Nbr*^cas9^ is a deletion allele. DNAs were from whole flies. Genotypes: control (5905), *Nbr*^cas9/*+*^ and *Nbr*^cas9/cas9^. (D) Small RNA northern blot confirms that *Nbr*^cas9^ is a loss-of-function allele. Whereas control flies expressed miR-34 normally, showing three major mature forms, *Nbr*^cas9^ flies lacked the smaller isoforms with an accumulation of the top isoform, reflecting a trimming defect. Expression of a wild-type *Nbr* transgene in *Nbr*^cas9^ flies restored all miR-34 isoforms, indicating a functional rescue. RNAs were from whole flies. Genotypes: control (5905), *Nbr*^cas9/cas^^9^ (*Nbr*^cas9^) and pUAST-*Nbr*, *Nbr*^cas9/cas9^; GeneSwitch-*tubulin*-GAL4 (*Nbr*^cas9^ *Nbr*).

To assess *Nbr* activity, we generated a new *Nbr* loss-of-function allele using the CRISPR/Cas9 method (Ren et al., 2013; Yu et al., 2013). We introduced two single guide RNAs (sgRNAs) that deleted 189 bp coding region for the exoribonuclease domain (Fig. 2A-C), hereafter termed *Nbr*^cas9^. Assessment of miR-34 isoforms confirmed *Nbr*^cas9^ as a null mutation (Fig. 2D). We characterized *Nbr*^cas9^ flies and identified miRNAs that were Nbr substrates (Fig. S2, Table S1).

**Fig. 3.**
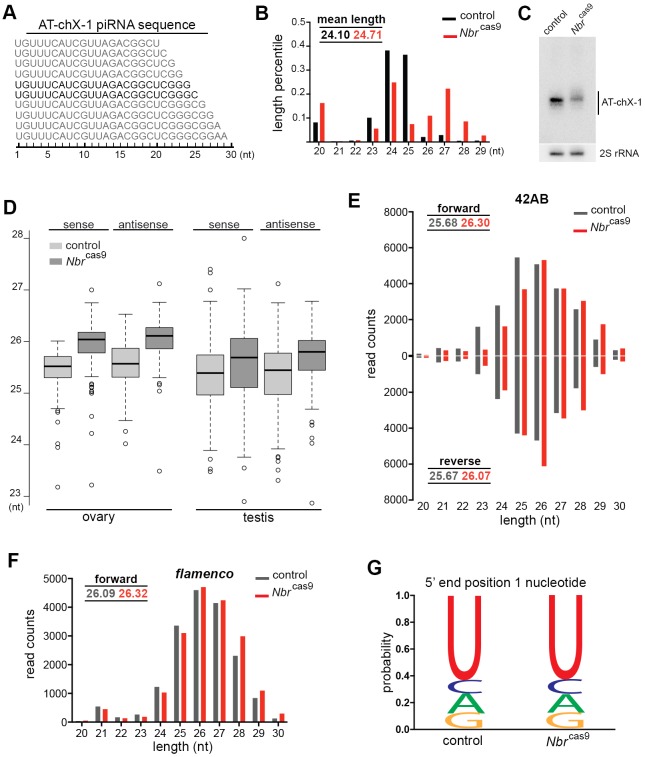
**Nbr trims piRNAs at the 3′ end.** (A) AT-chX-1, a piRNA enriched in the testis, displays the sequence feature of a 5′ uridine and a nested series at the 3′ end. The main forms are 24 and 25 nt (bold). (B) Length distribution analysis indicates that AT-chX-1 accumulates more long forms in *Nbr*^cas9^ (red) than in the control (black). RNAs were from testis. Genotypes: control (5905) and *Nbr*^cas9/cas9^ (*Nbr*^cas9^). (C) Small RNA northern blot confirms that AT-chX-1 shows a striking size increase in length in *Nbr*^cas9^ compared with control. 2S rRNA was used as a loading control. RNAs were from testis. (D) Gonadal piRNAs become longer in *Nbr* mutants than in controls. Box plots for length distribution revealed that *Nbr*^cas9^ flies accumulated more piRNAs with long forms than in controls. Using piPipes algorithms, only piRNAs mapped to transposons were used for subsequent analysis. Each transposon is assigned a mean value based on the length of mapped piRNAs, with a total of 127 individual transposons analyzed. Control versus *Nbr*^cas9^: ovary sense, *P*<2.2×10^−16^; ovary antisense, *P*<2.2×10^−16^; testis sense, *P*=4.861×10^−7^; testis antisense, *P*=1.19×10^−10^; Wilcoxon signed-rank test. RNAs were from ovaries and testis. (E,F) In the *42AB* cluster (E), piRNAs can be derived from both forward (top) and reverse (bottom) strands, and they show accumulation of long forms regardless of their strand origin. In the *flamenco* cluster (F), piRNAs can only be generated from the forward strand (top), and they show *Nbr*-dependent length modulation. Mean lengths for piRNAs derived from the indicated clusters in control and *Nbr*^cas9^ are shown. RNAs were from ovaries. (G) Antisense piRNAs in *Nbr*^cas9^ show the same bias for 5′ uridine (1U) as in the control. (C-G) Genotypes as in B.

To focus on piRNAs, we sequenced small RNAs from dissected germlines, comparing control with *Nbr*^cas9^. First, we analyzed the AT-chX-1 locus, a testis-enriched piRNA. In *Nbr* mutants, AT-chX-1 remained identical in sequence layout as in controls, but with a striking accumulation of longer forms as shown by length distribution and small RNA northern blot (Fig. 3A-C). Analysis of another well-characterized testicular piRNA derived from the *Su(Ste)* locus (Nishida et al., 2007) revealed a similar alternation at the 3′ end in *Nbr*-deficient gonads (Fig. S3).

Next, we carried out analysis on piRNA populations. We used a newly developed piPipes algorithm to plot the mean length of piRNA reads assigned to transposons (Han et al., 2015b). The rationale of this analysis lies in the fact that mutants would accumulate long or short forms depending on the nature of defects, thus causing the mean length of piRNAs to depart from that of controls (Fig. S4). We found that loss of *Nbr* had a global impact on the length distribution of piRNAs with a clear accumulation of longer forms, regardless of their strand origins (Fig. 3D). We then characterized the impact of *Nbr* deletion on two specific piRNA clusters: *42AB* piRNAs, which are bidirectional transcripts in germline cells, and *flamenco* piRNAs, which are prominently unidirectional transcripts in follicle cells of somatic tissues (Brennecke et al., 2007). The loss of *Nbr* led to a size increase in both populations of piRNAs (Fig. 3E,F), suggesting that Nbr is likely to regulate piRNA sequences after their transcription with minimal strand preference. To determine if the size increase in gonadal piRNAs in *Nbr*^cas9^ flies is due to a lack of nucleotide removal on either the 5′ or 3′ end, or both, we carried out an analysis on antisense piRNAs that have 1U bias (Brennecke et al., 2007). Comparing control with *Nbr* mutants, we found unaltered 1U bias of piRNAs (Fig. 3G), suggesting that the size increase is unlikely to be due to changes at the 5′ end.

Taken together, these results demonstrated that the increase in piRNA length in *Nbr* mutants is primarily due to the accumulation of forms with extended 3′ ends, indicative of a failure in 3′ processing upon *Nbr* depletion. Since Nbr has an established 3′-to-5′ exoribonuclease activity in trimming small RNAs (Han et al., 2011), these results, together with its protein-protein interaction with Piwi, demonstrate an *in vivo* biological role of Nbr in trimming piRNAs from germline and somatic tissues.

### Genome-wide impact of *Hen1* depletion at 3′ ends of piRNAs

**Fig. 4.**
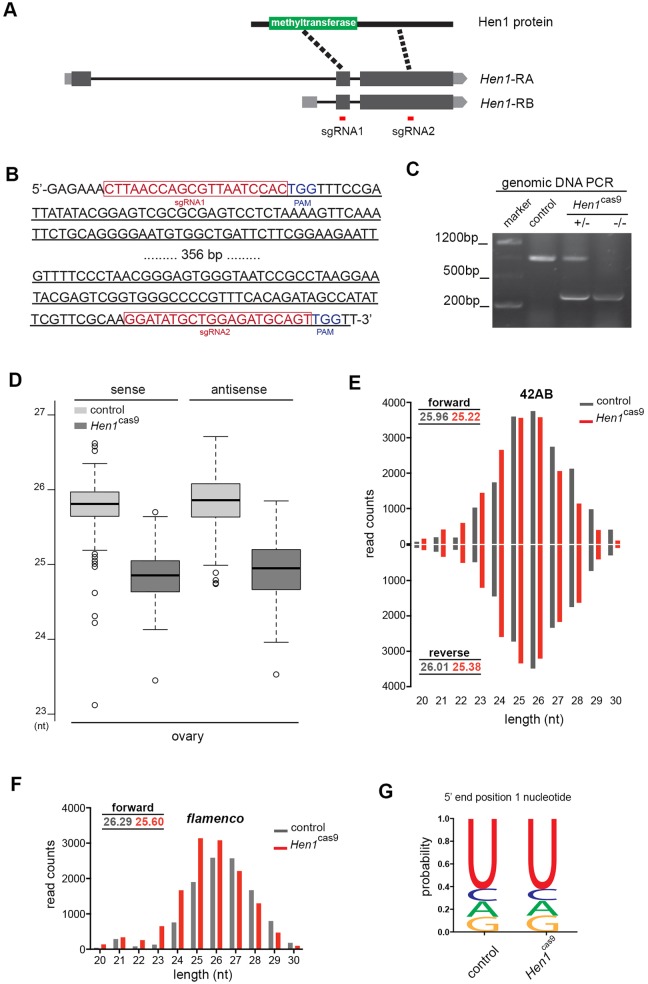
**Loss of *Hen1* shortens piRNAs from the 3′ end.** (A) The *Hen1* locus and guiding RNAs used to make *Hen1*^cas9^. To produce the *Hen1* loss-of-function allele, sgRNA1 and sgRNA2 (red lines) were used to induce site-specific deletion. The deleted region largely removed the methyltransferase domain. (B) Part of the *Hen1* genomic sequence illustrating features of the CRISPR/Cas9-mediated *Hen1*^cas9^ loss-of-function allele. sgRNAs and PAMs are highlighted. Resulting *Hen1* mutant flies were backcrossed to the control homogeneous background for five generations to ensure background clearance. (C) PCR analysis confirms that *Hen1*^cas9^ is a deletion allele. DNAs were from whole flies. Genotypes: control (5905), *Hen1*^cas9/*+*^ and *Hen1*^cas9/cas9^. (D) Box plots for length distribution reveal that *Hen1*^cas9^ flies accumulate more short form piRNAs than controls. Control versus *Hen1*^cas9^: ovary sense, *P*<2.2×10^−16^; ovary antisense, *P*<2.2×10^−16^; Wilcoxon signed-rank test. RNAs were from ovaries. Genotypes: control (5905) and *Hen1*^cas9/cas9^ (*Hen1*^cas9^). (E,F) In the *42AB* cluster (E) and the *flamenco* cluster (F), piRNAs show accumulation of shorter forms. Mean lengths for piRNAs derived from the indicated clusters in control and *Hen1*^cas9^ are shown. RNAs were from ovaries. (G) Antisense piRNAs in *Hen1*^cas9^ show the same bias for 5′ uridine (1U) as in control. RNAs were from ovaries. (E-G) Genotypes as in D.

Hen1, a methyltransferase, is known to be involved in piRNA pathways by catalyzing 2′-*O*-methylation at the 3′ terminal nucleotide (Horwich et al., 2007; Saito et al., 2007). Yet, how Hen1 activity impacts the piRNA profile at the genome level remains uncharacterized. To explore its role, we generated a new *Hen1* loss-of-function allele using the CRISPR/Cas9 method (Fig. 4A-C), hereafter termed *Hen1*^cas9^. Following the same rationale, we sequenced and analyzed piRNAs from ovaries of control and *Hen1* mutants. Interestingly, we found that the lack of *Hen1* led to a dramatic shortening of piRNA from the 3′ end (Fig. 4D-G). Of note, our data provide the first insight at the genome level into the role of *Hen1*. Since depletion of *Hen1* results in a piRNA profile characterized by enhanced 3′ trimming, we proposed that Hen1-mediated 2′-*O*-methylation might play a role in antagonizing trimming at piRNA 3′ ends.

### Antagonistic roles of *Nbr* and *Hen1* modulate piRNA 3′ ends

**Fig. 5.**
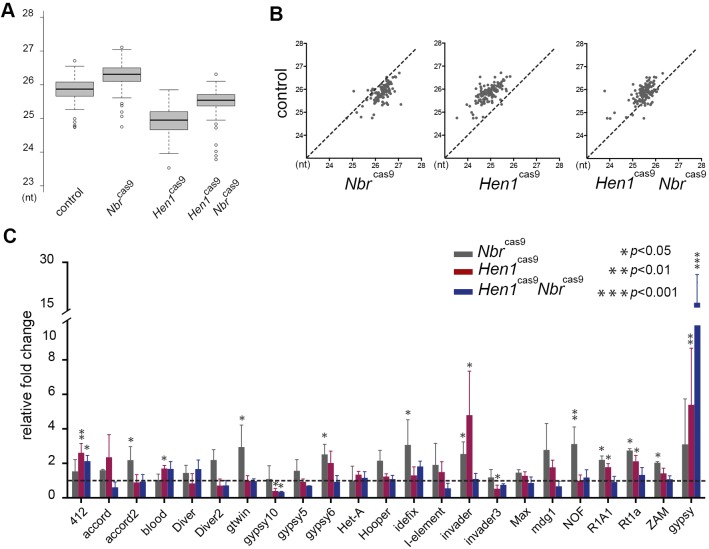
**Antagonistic roles of *Nbr* and *Hen1* in piRNA pathways.** (A,B) Box plots (A) and scatter plots (B) showing that *Nbr* and *Hen1* modulate piRNA length. Whereas lack of *Nbr* lengthened piRNAs, lack of *Hen1* shortened them. Flies lacking both *Nbr* and *Hen1*, however, showed restoration of the piRNA profile towards that of controls. *Nbr*^cas9^ versus control (5905), *P*<2.2×10^−16^; *Hen1*^cas9^ versus control, *P*<2.2×10^−16^; *Hen1*^cas9^ *Nbr*^cas9^ versus control, *P*<2.2×10^−16^; Wilcoxon signed-rank test. piRNA sequencing and analysis of RNA from ovaries of 3-day-old animals of the indicated genotypes. Genotypes: *Nbr*^cas9/cas9^ (*Nbr*^cas9^), *Hen1*^cas9/cas9^ (*Hen1*^cas9^) and double mutant. (C) *Nbr* and *Hen1* are functionally relevant in repressing TEs. qRT-PCR analysis was used to determine the expression status of TEs among different genotypes. Whereas lack of either *Nbr* or *Hen1* caused upregulation of select transposons, flies mutant for both *Nbr* and *Hen1* exhibited a clear reversion in that the majority of the desilenced TEs became repressed, the only exceptions being *412* and *gypsy*. Mean±s.d., *n*=3 independent experiments; Student's *t*-test. RNAs were from ovaries. Genotypes as in A.

To determine a potential interaction of Hen1 and Nbr in 3′ trimming of piRNAs, we sequenced and analyzed piRNAs from control, *Nbr*^cas9^, *Hen1*^cas9^, and *Hen1*^cas9^ *Nbr*^cas9^ double-mutant flies. Consistently, this analysis demonstrated that whereas *Nbr*^cas9^ lengthened piRNAs, *Hen1*^cas9^ shortened them (Fig. 5A,B, Table S2). Moreover, we found that flies carrying the *Hen1*^cas9^ *Nbr*^cas9^ double mutation showed a rescuing effect on the overall piRNA length profile towards that of controls (Fig. 5A,B, Table S2). Intriguingly, an oxidation and β-elimination experiment revealed that piRNAs remained 2′-*O*-methylated in *Nbr*^cas9^ mutants, suggesting that Nbr-mediated trimming is not required for methylation to take place at piRNA 3′ ends (Fig. S5). None of these mutants affected the expression of core piRNA pathway factors (data not shown). piRNA abundance appeared to be at comparable levels between controls and the various mutants (Fig. S6A, Table S3). Combined, these data suggested antagonizing roles of *Nbr* and *Hen1* in modulating piRNA 3′ ends.

### Silencing of select TEs is disturbed upon loss of either *Nbr* or *Hen1*

Since a major function of mature piRNAs in animal gonads is for silencing aberrant TE expression (Aravin et al., 2007), we next asked if Nbr and *Hen1* activity might affect TE silencing. Mutations in genes that encode the core factors of piRNA pathways, such as *piwi*, *aub* and *Ago3*, lead to strong defects in fertility. Despite the shortened piRNAs, *Hen1* mutants show normal fertility, suggesting that lack of *Hen1* does not cause a strong defect in the germline (Fig. S6B). By contrast, *Nbr*^cas9^ mutants and *Hen1*^cas9^ *Nbr*^cas9^ double mutants had reduced fecundity (Fig. S6B).

To characterize the TE status in *Nbr*^cas9^ and *Hen1*^cas9^ flies, we performed qRT-PCR analysis of ovary RNAs. The selection of TEs was based upon the existing literature that characterized representative TEs across the genome (Li et al., 2009). This analysis revealed that, among 23 TEs analyzed, eight in *Nbr*^cas9^ and six in *Hen1*^cas9^ showed loss of silencing (Fig. 5C). In *Hen1*^cas9^ *Nbr*^cas9^ double mutants, more interestingly, we observed a striking trend in that a majority of desilenced TEs (10 out of 12 upregulated in the single mutant) were now brought back to normal silenced status, with the only exceptions being *412* and *gypsy* (Fig. 5C). piRNAs mapped to *412* and *gypsy* transposons behaved as expected in control and the various mutant backgrounds (Tables S2 and S3), so modulation of *412* and *gypsy* cannot be attributed to alteration of the piRNA profile per se, but rather influenced by other effects in *Hen1*^cas9^ *Nbr*^cas9^ double mutants. Since *Nbr* is involved in both miRNA and piRNA pathways, the biological consequence of double mutants might in part be contributed by defective trimming of select miRNAs. Nevertheless, our data indicated that the proper control of TE silencing could be disturbed upon the loss of either *Nbr* or *Hen1*. Importantly, rescue of TE silencing in the double mutant was accompanied by a restoration of piRNA length, suggesting a regulatory mode that is sensitive to piRNA length. Since Nbr and Hen1 have opposing effects on piRNA length, our data seemed to suggest that the integrity of piRNAs, including their length, is essential to maintain the normal repression status of most TEs.

### piRNAs are age modulated

**Fig. 6.**
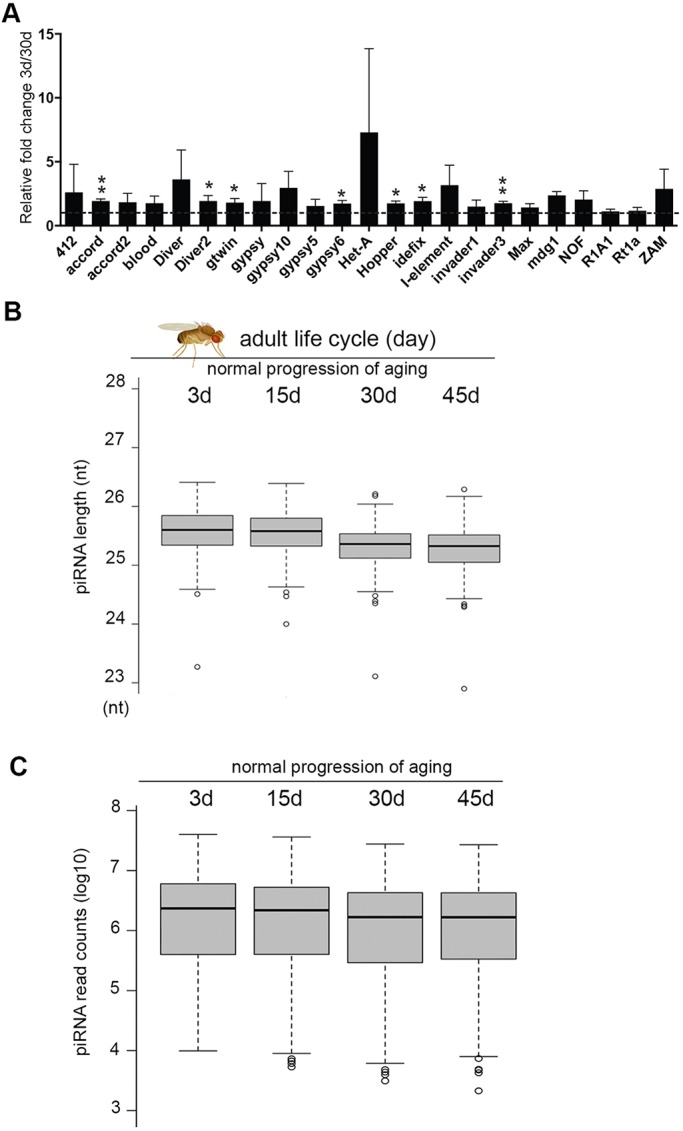
**piRNAs are age modulated.** (A) Select transposons are upregulated in aged ovaries. qRT-PCR analysis was used to determine the expression status of TEs in ovaries at 30 days versus 3 days. Mean±s.d., *n*=3 independent experiments; *P<0.05, **P<0.01, Student's *t*-test. (B) The length of piRNAs is age modulated. RNA from ovaries of control flies at 3 (newly eclosed adult), 15 (young), 30 (mid-age) and 45 (old) days of age were used to generate age-modulated piRNA profiles. Box plots show that with age piRNAs become progressively shortened from the 3′ end. Fifteen versus 3 days, *P=*0.001371; 30 versus 3 days, *P*<2.2×10^−16^; 45 versus 3 days, *P*<2.2×10^−16^; Wilcoxon signed-rank test. (C) The abundance of piRNAs in ovaries becomes slightly decreased with age. Box plots for normalized read counts are shown as log_10_ value. Fifteen versus 3 days, *P=*3.182×10^−1^; 30 versus 3 days, *P*<2.2×10^−16^; 45 versus 3 days, *P*<2.2×10^−16^; Wilcoxon signed-rank test. (A-C) Flies were of genotype 5905.

A fertility test revealed that flies demonstrate an age-associated decline in fecundity (Fig. S7A); yet, few studies have addressed whether late-onset fertility decline is coupled with an age modulation of TE status and piRNAs. To approach these issues, we first characterized the expression profile of TEs during aging. We found that aged flies demonstrated significant derepression of select TEs compared with young animals (Fig. 6A). Next, we asked if the piRNA profile was also modulated during aging. We sequenced piRNAs from ovaries of control flies at 3 (newly eclosed adult), 15 (young), 30 (mid-age) and 45 (old) days of age (Fig. 6B). Since our control isogenic flies had a median life span of ∼60-70 days, we avoided testing animals at 60 days to uncouple potential defects with lethality. Interestingly, during normal aging, piRNAs appeared to undergo a progressive shortening from the 3′ end (Fig. 6B, Fig. S7B, Table S4). Coupled with the shortening, piRNA abundance also showed a slight decline with age (Fig. 6C, Table S5). Although the change in abundance is very minor, it is statistically significant. Together, these data provided a first glimpse into the piRNA profile of the adult life cycle and revealed a potential link between the age-modulated piRNA profile and a deterioration of TE silencing status with age.

### *Nbr* contributes to the age-modulated piRNA profile

**Fig. 7.**
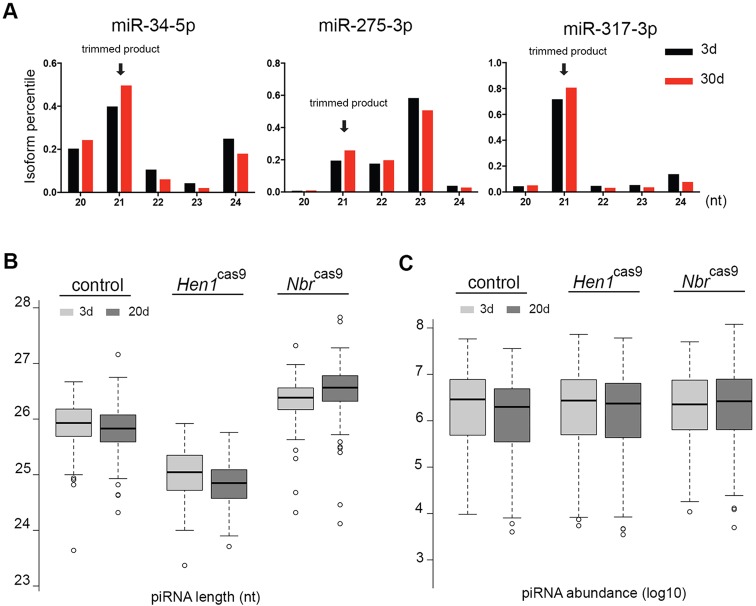
**The age modulation of piRNAs is dependent on Nbr activity.** (A) Nbr trimming activity increases with age. Small RNA deep sequence datasets were used. Known Nbr-trimmed isoforms (arrows) of miR-34-5p, miR-275-3p and miR-317-3p show increased levels at 30 days compared with 3 days. RNAs were from ovaries of genotype 5905. (B) *Nbr* contributes to age modulation of piRNA length. Box plots show that with aging piRNAs become shortened in control and *Hen1*^cas9^, but not in *Nbr*^cas9^. Twenty versus 3 days: control (5905), *P*=2.948×10^−10^; *Hen1*^cas9^, *P=*2.634×10^−15^; *Nbr*^cas9^, *P=*7.098×10^−8^; Wilcoxon signed rank test. *Nbr*^cas9/cas9^ (*Nbr*^cas9^), *Hen1*^cas9/cas9^ (*Hen1*^cas9^). (C) *Nbr* modulates piRNA abundance with age. Box plots showed a trend in that with aging piRNA levels were decreased in control and *Hen1*^cas9^, but not in *Nbr*^cas9^. Twenty versus 3 days: control (5905), *P*<2.2×10^−16^; *Hen1*^cas9^, *P=*8.454×10^−11^; *Nbr*^cas9^, *P=*0.1271; Wilcoxon signed-rank test. Genotypes as in B.

Age modulation of piRNAs showed a uniform pattern of change, suggesting a controlled event rather than dysregulated activities of *Nbr* or *Hen1* upon aging. Using qRT-PCR, we found that the steady-state levels of both *Nbr* and *Hen1* increased in aging ovaries (Fig. S8A). In *Nbr*^KI-Myc^ flies, Nbr protein levels remained roughly the same between 3 and 30 days (Fig. S8B). To test the possibility that the reduction in piRNA length at the 3′ end during aging was due to enhanced trimming by Nbr, we compared the profile of known miRNA substrates of Nbr in young and old flies. Interestingly, the levels of miR-34-5p shorter isoforms, which are established Nbr trimming products, were markedly increased in aged ovaries (Fig. 7A). Similar changes were also found for miR-275-3p and miR-317-3p, with their Nbr-dependent isoforms being elevated with age (Fig. 7A), which was consistent with the increased activity of Nbr with age.

To further determine if Nbr is involved, we sequenced piRNAs from control, *Nbr*^cas9^ and *Hen1*^cas9^ animals of 3 or 20 days of age. Day 20 was chosen to uncouple the onset of age-associated phenotypes in *Nbr* (current study) or *Hen1* (Abe et al., 2014) mutants. Strikingly, our data revealed that whereas piRNAs displayed age-associated shortening in control and *Hen1*^cas9^, piRNAs in *Nbr*^cas9^ did not (Fig. 7B, Fig. S8C). The abundance of piRNAs also showed a slight but statistically significant decline between 20 days and 3 days in control and *Hen1*^cas9^, but not in *Nbr*^cas9^ (Fig. 7C, Fig. S8D). Together, these data implicated an age-associated activity of Nbr leading to enhanced trimming, which in turn contributes to the age-modulated piRNA profile as characterized by a gradual shortening as well as a decline in abundance.

## DISCUSSION

**Fig. 8.**
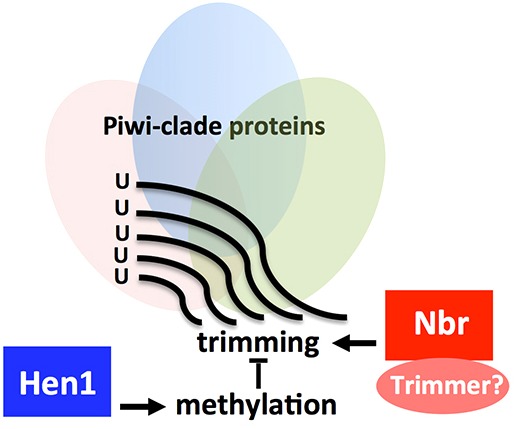
**Model depicting antagonistic roles of *Nbr* and *Hen1* in the modulation of piRNA 3′ ends.** There is a delicate balance in the mechanism by which *Nbr* and *Hen1* modulate piRNA 3′ ends. Mature piRNAs of ∼23-30 nt are bound to PIWI clade Argonaute proteins (ovals), including Piwi, a nodal protein that is crucial in piRNA pathways. We provide evidence that Piwi can interact with Nbr. Our data reveal interplay between Nbr-mediated exoribonuclytic trimming and Hen1-dependent methylation that antagonizes trimming, thus linking two genes with opposing activity in shaping piRNA 3′ ends in the biogenesis of piRNAs. Nbr might represent just one of a large cohort of additional, as yet unknown, factors with roles in trimming piRNA 3′ ends.

A long-standing question in piRNA pathways is to understand the biogenesis and modulation of mature piRNAs. To date, a spectrum of protein factors has been implicated in piRNA biogenesis pathways; by contrast, few genes have been carefully studied in the modulation of mature piRNAs. Here, we provide evidence to suggest a model for the modulation of piRNA 3′ ends (Fig. 8). First, piRNA substrates longer than the mature length are loaded onto the Piwi protein. Nbr, an established 3′-to-5′ exoribonuclease, mediates trimming until Hen1 catalyzes the 2′-*O*-methylation on mature piRNAs of proper length. Yet, our data implicate that trimming and methylation are not coupled events. Mature piRNAs of proper length and 3′ end modification can be readily enrolled into piRNA pathways. Within this model, there is a delicate balance in the interplay of Nbr and Hen1: genetic mutation abolishing either component overwhelmingly alters the balance, diminishing the efficacy of piRNA pathways; on the other hand, enhancement of one player over the other, such as an increased activity of Nbr with age, gradually tilts the balance, altering piRNAs in response to the progression of natural aging. Intriguingly, flies with loss of both *Nbr* and *Hen1* show shorter piRNAs than with loss of *Nbr* alone (Fig. S9), implicating a possible effect of a second, as yet unknown, ‘trimmer’. As such, Nbr and Hen1 might represent examples of a group of emerging factors crucial for the modulation of piRNA sequences.

Nbr and Hen1 might define one key aspect shared by additional factors in shaping piRNA 3′ ends. Previous studies identified Papi, a Tudor domain protein that can influence piRNA length (Han et al., 2015a; Honda et al., 2013). Yet, analysis of the Nbr interactome based on an endogenous protein bait and a rigorous filter only captured Piwi, a signature protein of piRNA pathways, but not Hen1 and Papi. These data might suggest that these factors do not necessarily form a protein complex but instead mediate parallel processes in the modulation of piRNA 3′ ends. Although we have shown protein interactions between Nbr and Piwi, these results do not exclude the possibility that Nbr may function together with Aub or Ago3 through transient or dynamic interactions. Alternatively, rather than being direct contact, adapter proteins might be necessary to bridge Nbr with other PIWI family members. Importantly, our data clearly show an effect of Nbr in trimming of AT-chX-1, a testicular piRNA bound to Aub but not Piwi protein (Nishida et al., 2007). Therefore, a detailed mechanism remains to be elucidated by which Nbr can differentially trim piRNAs bound to distinct PIWI family proteins. Furthermore, it is worth noting that Piwi protein can shuttle between the nucleus and cytoplasm, whereas Nbr is primarily cytoplasmic (Feltzin et al., 2015). Thus, Nbr activity can only partially overlap with that of Piwi.

Fertility is an adult-onset trait, which can progressively decline in old age. Interestingly, strong mutations that abolish piRNA pathways are always coupled with a severe fertility defect, indicating an underlying connection between the integrity of piRNAs and the maintenance of adult fertility. Here, we provide evidence that in the adult life cycle piRNAs undergo a progressive shortening from the 3′ end and a decline in abundance, striking age-related features that have not been previously demonstrated. Furthermore, our data support the activity of Nbr in the promotion of the piRNA aging profile, indicating an active mode of trimming that proceeds along with the progression of aging. Nevertheless, aging is a highly complex biological event that converges effects from multiple intrinsic drivers as well as the environment (Kirkwood, 2005). Our data that link Nbr trimming with the chronic modulation of piRNAs present a novel facet in the understanding of age-associated events. Future studies may address how this link might impact the long-term maintenance of TEs and fertility in the adult life cycle.

## MATERIALS AND METHODS

### Fly genetics

Flies were grown at 25°C. To make *Nbr*^cas9^ flies, we followed the method developed by Yu et al. (2013). To make the *Hen1*^cas9^ and *piwi*^cas9^ mutants, we used the method developed by Ren et al. (2013). To make *Nbr*^KI-Myc^ flies, we first recombined the *Lig4*^−/−^ mutant with flies expressing *nano*-Cas9 (TH00788. N), followed by microinjection of sgRNA and the oligonucleotide template with the Myc tag sequence. Fly microinjection was conducted by the *Drosophila* Core Facility, Institute of Biochemistry and Cell Biology, Chinese Academy of Sciences. To induce *Nbr* transgene expression in adults, we recombined *Nbr* pUAST transgenic flies with the *Tubulin*-*GeneSwitch*-GAL4 line, and fed adult flies with standard media containing 100 μg/ml Mifepristone (Sigma). Further details of strain construction are provided in the supplementary Materials and Methods.

Fly wing posture phenotype was scored at days 3 and 10. For each genotype at each time point, 40 flies were scored. Three independent experiments for each genotype and time point were performed for statistics. To determine lifespan, newly eclosed males were maintained at 15 flies per vial, transferred to fresh vials every 2 days while being scored for survival. A total of 150-200 flies were used per genotype per lifespan. The spreadsheet for the lifespan was first generated in Excel (Microsoft) and then analyzed by Prism software (GraphPad) for survival curves and statistics. To determine female fecundity, we crossed three healthy wild-type males with one virgin female of defined genotype or age. The crosses lasted for 48 h, and then adult flies were removed. After an additional 10 days, emerged flies of the next generation were counted. For each genotype, ten independent virgin females were scored.

### Protein immunoprecipitation and mass spectrometry

Adult virgin female flies of control and *Nbr*^KI-Myc^ were collected and aged to 3 days. 400 ovaries for each genotype were dissected and homogenized in 1 ml lysis buffer [50 mM Tris-HCl pH 7.5, 5% glycerol, 0.4% NP40, 1.5 mM MgCl_2_, 125 mM NaCl, 25 mM NaF, 1 mM Na_3_VO_4_, 1 mM DTT, 1 mM EDTA and Complete Protease Inhibitor (Roche)] using a pre-cooled Dounce tissue homogenizer. The homogenates were centrifuged at 12,000 ***g*** for 5 min at 4°C. The supernatants were passed through a 100 μm cell strainer followed by 0.45 μm PVDF membrane. 100 μl anti-c-Myc agarose beads (Sigma) were washed with 1 ml TBS buffer (50 mM Tris HCl, 150 mM NaCl, pH 7.4) and then mixed with protein extracts. After a 2 h incubation at 4°C, beads were washed three times with 1 ml TBS buffer. After the final wash, beads were dissolved in 60 μl 8 M urea, 100 mM Tris pH 8.5, and then reduced by 5 mM Tris (2-carboxyethyl) phosphine for 20 min and alkylated by 10 mM iodoacetamide for 15 min in the dark. The solution was then diluted 1:4 with 100 mM Tris pH 8.5, and digested with 1 μg trypsin at 37°C overnight. The digestion was terminated by adding 2% formic acid, and subjected to C18 desalting tips. The resulting peptides were dried in a vacuum concentrator. The peptides were analyzed by online nanoflow liquid chromatography tandem mass spectrometry (LC-MS/MS). Briefly, the peptides were first separated on a nano column (100 μm×15 cm, C18, 1.9 μm, 120 Å) using an EASY-nLC 1000 system (Thermo Scientific). The separated peptides were analyzed using an Orbitrap Fusion mass spectrometer (Thermo Scientific). A cycle of one full-scan mass spectrum (300-1800 m/z) at a resolution of 120,000 followed by higher-energy collisional dissociation-ion trap (HCD-IT) MS/MS spectra at 32% normalized collision energy was repeated continuously in top-speed mode. The mass spectrometry data were analyzed by Proteome Discoverer (Thermo Scientific, ver. 1.4). The tandem mass spectra were searched against the UniProt *Drosophila melanogaster* protein database (release date: 27/05/2015) using the Sequest HT search engine (Eng et al., 1994). Carbamidomethyl of cysteine was set as a static modification, and dynamic modification of methionine oxidation was used. The peptide false discovery rate was controlled at 1% by Percolator (Kall et al., 2007). Peptide-spectrum match (PSM) numbers of corresponding proteins were used to assess differences in protein abundance.

### Small RNA sequencing

Fly tissues were dissected in ice-cold 1× phosphate buffered saline solution (Sangon Biotech), and total RNA was isolated using Trizol according to the manufacturer's instructions (Life Technologies). DNA was removed using the Ambion TURBO DNA-free Kit (Life Technologies). RNA was fractionated using 15% TBE-urea pre-cast PAGE gels (Life Technologies). Small RNA of ∼20-29 nt was sliced from the gel and recovered using the ZR Small-RNA PAGE Recovery Kit (Zymo Research). To determine library quality, qRT-PCR was used to quantify the library concentration. The normalized libraries were denatured with 0.1 M NaOH solution (Sigma). Pooled libraries with different barcodes were sequenced on the Illumina Miseq platform.

### Sequence analysis

Adapter sequence was removed using FASTX (http://hannonlab.cshl.edu/fastx_toolkit/). Reads without adapter, shorter than 18 nt or mapped to rRNAs were filtered out. For annotating miRNAs, we used miRDeep2 (Friedlander et al., 2008). *Drosophila* genome release 6 from FlyBase was used as reference, and the miRNA precursor and mature miRNAs were from miRBase. miRNA isoform statistics were carried out according to miRDeep2 output, and reads were restricted with perfect match. For annotating piRNAs, small RNA reads were mapped to transposon reference sequence downloaded from FlyBase using Bowtie2 (Langmead and Salzberg, 2012) with two mismatches allowed. Probability for 5′ nucleotide was calculated by FASTX. Length distribution and graphic analysis of piRNAs derived from different transposons were performed using the piPipes algorithm (Han et al., 2015b). Scatter plots were produced using piPipes. Box plots were produced using R language, according to piPipes output. To calculate piRNA abundance, reads was first normalized to siRNAs to attain normalized counts. Dm3 was chosen as genomic reference and two mismatches were allowed for mappings.

### Small RNA northern

For small RNA northern, total RNA was isolated from fly head, whole flies or fly ovaries using Trizol reagent as above. For each lane, 3 μg RNA was fractionated on 15% TBE-urea pre-cast PAGE gels (Life Technologies), and then transferred onto a Hybond nylon membrane (GE Healthcare). Prior to hybridization, RNA blots were prehybridized with Ambion Oligohyb (Life Technologies), and then incubated with radioactively labeled RNA probes made using the Ambion Maxiscript-T7 *In Vitro* Transcription Kit (Life Technologies), supplemented with ^32^P-labeled UTP. Oligonucleotide templates were prepared by annealing two single-stranded DNA oligonucleotides into a duplex (99°C for 5 min and then allowed to cool to room temperature). Probes are listed in the supplementary Materials and Methods.

### Molecular biology

To clone full-length cDNA, RT-PCR was conducted using RNAs from ovaries. Primers are listed in the supplementary Materials and Methods.

To carry out *Drosophila* Schneider 2 cell-based co-IP experiments, 8×10^6^ cells were seeded onto a 10 cm plate, and plasmid DNAs were transfected using Effectene (Qiagen). Cells were collected 36 h after transfection and lysed in lysis buffer (as above) for 30 min on ice. Lysates were centrifuged 12,000 ***g*** at 4°C for 5 min, and the supernatants were transferred to new tubes. 10% supernatants were used as input. Protein A/G PLUS-agarose (Santa Cruz) was added for preclear at 4°C for 1 h. The anti-Flag antibody (1:1000; Sigma, F1894) was added and incubated at 4°C for 2 h. Then protein A/G PLUS-agarose was added. Flag-tagged proteins were immunoprecipitated overnight at 4°C. Beads were washed three times with RIP buffer (150 mM KCl, 25 mM Tris pH 7.4, 5 mM EDTA, 0.5 mM DTT, 0.5% NP40). For western blots, 15 adult male heads, five adult muscles, or five adult ovaries per sample were homogenized in 50 μl Laemmli buffer (Bio-Rad) supplemented with 5% 2-mercaptoethanol, heated to 95°C for 5 min and 10 μl was loaded onto 10% bis-Tris gels (Life Technologies), then transferred to nitrocellulose membrane (Bi-Rad) and blotted following standard protocols. Primary antibodies used were anti-Flag (1:1000; Sigma, F1894), anti-HA (1:2000; Roche, 12013819001), anti-Tubulin (1:10,000; MBL, PM054) and anti-Myc (1:2000; Santa Cruz Biotechnology, sc-40). Secondary antibodies were goat anti-rabbit (1:5000; Sigma, A9169) and goat anti-mouse (1:5000; Sigma, A4416) conjugated to HRP, then developed by chemiluminescence (ECL, Thermo Scientific). The final image was obtained by Fuji scanner (GE Healthcare). To make the Piwi polyclonal antibody, 200 amino acids of the N-terminus of the Piwi protein were used as antigen and injected into rabbit according to established methods (GenScript).

To determine transposon expression status, we performed qRT-PCR using RNA from fly ovaries. The analysis was performed using the QuantStudio 6 Flex real-time PCR system (Life Technologies).

### Dataset access

All sequence datasets have been uploaded into the Sequence Read Archive (SRA, http://trace.ncbi.nlm.nih.gov/Traces/) with access ID SRP062894.
